# New insights on the Ife-Ilesha schist belt using integrated satellite, aeromagnetic and radiometric data

**DOI:** 10.1038/s41598-021-94813-1

**Published:** 2021-07-28

**Authors:** Naheem Banji Salawu, Julius Ogunmola Fatoba, Leke Sunday Adebiyi, Muyiwa Michael Orosun, Silas Sunday Dada

**Affiliations:** 1 BS Geophysical and Consultancy Ltd., Ilorin, Nigeria; 2 Department of Geophysics, Federal University Oye-Ekiti, Ekiti, Nigeria; 3 Department of Physical Sciences, Landmark University, Omu‑Aran, Nigeria; 4 Department of Physics, University of Ilorin, Ilorin, Nigeria; 5 Dasil Geoconsults & Co., Ilorin, Nigeria; 6 Department of Earth Sciences, Anchor University, Ayobo-Lagos, Nigeria

**Keywords:** Geology, Geomorphology, Geophysics, Mineralogy

## Abstract

The present study combined analysis of satellite, aeromagnetic and radiometric data for evaluation of structural features within the Ife-Ilesha schist belt. Shuttle Radar Topographic Mission digital elevation data have been enhanced using hill-shading technique for the delineation of morphological features. The superposition of total gradient amplitude lineaments on the 3-D Euler deconvolution map revealed the trends and depth of structural features within the study region. The major trends are NE–SW, NNE–SSW, E–W and minor trends in the N–S direction, including the Ifewara shear zone that trends in the NNE–SSW. The estimated depths to the top of the sources within the shear zone varies from 90 to 200 m. Complementary analysis of the airborne radiometric data revealed that the Ifewara shear zone and adjacent regions are characterized by radiometric anomalies, indicating regional mineralization alteration zone. Generally, there is a good correlation between the satellite, radiometric, aeromagnetic maps which provides new insights and re-evaluation of structural features.

## Introduction

Solid minerals are important components in the generation of modern technology, and can also help generate huge funds for a nation. Mineral deposits such as tin, tantalite and gold mineralization are naturally occurring resources, which are closely associated with structural features such as geological contacts, fractures, veins, shear zones, and faults within the Ife-Ilesha schist^1,2^. Generally, the nature of mineralization varies from primary (disseminated and quartz veins) to secondary (eluvial and alluvial) types.

Eluvial and alluvial placer concentrations that form the basis of artisanal activities are located close to the source rocks due to the high degree of chemical and physical weathering under the prevailing tropical conditions^1^. The disintegration of the source rock units combined with seasonal streams transportation has resulted in artisanal miners excavating random tin-tantalite-gold–quartz reefs. The artisanal miners’ activities predisposed numerous non-mineralized locations resulting in large scale land degradation with observable loss of land for agricultural purposes^3^. Mining of structurally controlled mineral deposits is hampered by scarcity of surface and subsurface structural data that can be generated by integrating satellite and geophysical data. Hence, the present geophysical investigation is conducted to update the structural map of the region to locate new mining activities. The structural map should serve as future guide for mineral exploration programs and can also help address the issue of indiscriminate excavation activities within the Ife-Ilesha schist belt. The observable structural features of the schist belt are not well exposed because of the humid tropical climate settings of the region in contrast to the Kalangai-Zungeru belt in the Savanna zone to the north. This makes satellite data suitable for mapping mineralized surface lineaments that are obscure by forest vegetation. Remote sensing technique has been utilized widely for delineation and analyses of surface lineament, lithological units, and topographical features associated with mineralization. The integration of aeromagnetic and radiometric data has proven particularly valuable in the examination of concealed structural features in mainly unexposed terrain such as the study area. Therefore, integrating satellite, aeromagnetic and radiometric data are very significant for the assessment of surface and subsurface lineaments that could host rich mineral deposits. A recent study conducted by Salawu et al.^3^ highlighted the effects of subsurface structures on surface lineaments with implications on the control of gold deposits around the Middle Niger Basin in Nigeria. Similarly, Salawu et al.^4^ integrated satellite and aeromagnetic data to investigate lineaments for the determination of mineral distribution in older E–W trending structures within southwestern Nigeria to further investigate the geological lineaments that can add to the understanding of structurally controlled mineralization within the Ife-Ilesha schist belt and associated basement.

There are different previous studies based on geochemistry, geological, remote sensing and geophysical aspects for understanding crustal evolution and tectonic studies over the Ife-Ilesha schist belt (e.g.^5–10^). However, there is hardly any integrated study for appraisal of mineral deposits within the Ife-Ilesha schist belt and its surroundings. Hence, this present study attempts to investigate the geological structures of the Ife-Ilesha schist belt by integrating radiometric, aeromagnetic and satellite data. This is to provide valuable information for the identification of potential economic mineral zones based on the structural features.

## The study area

**Figure 1:**
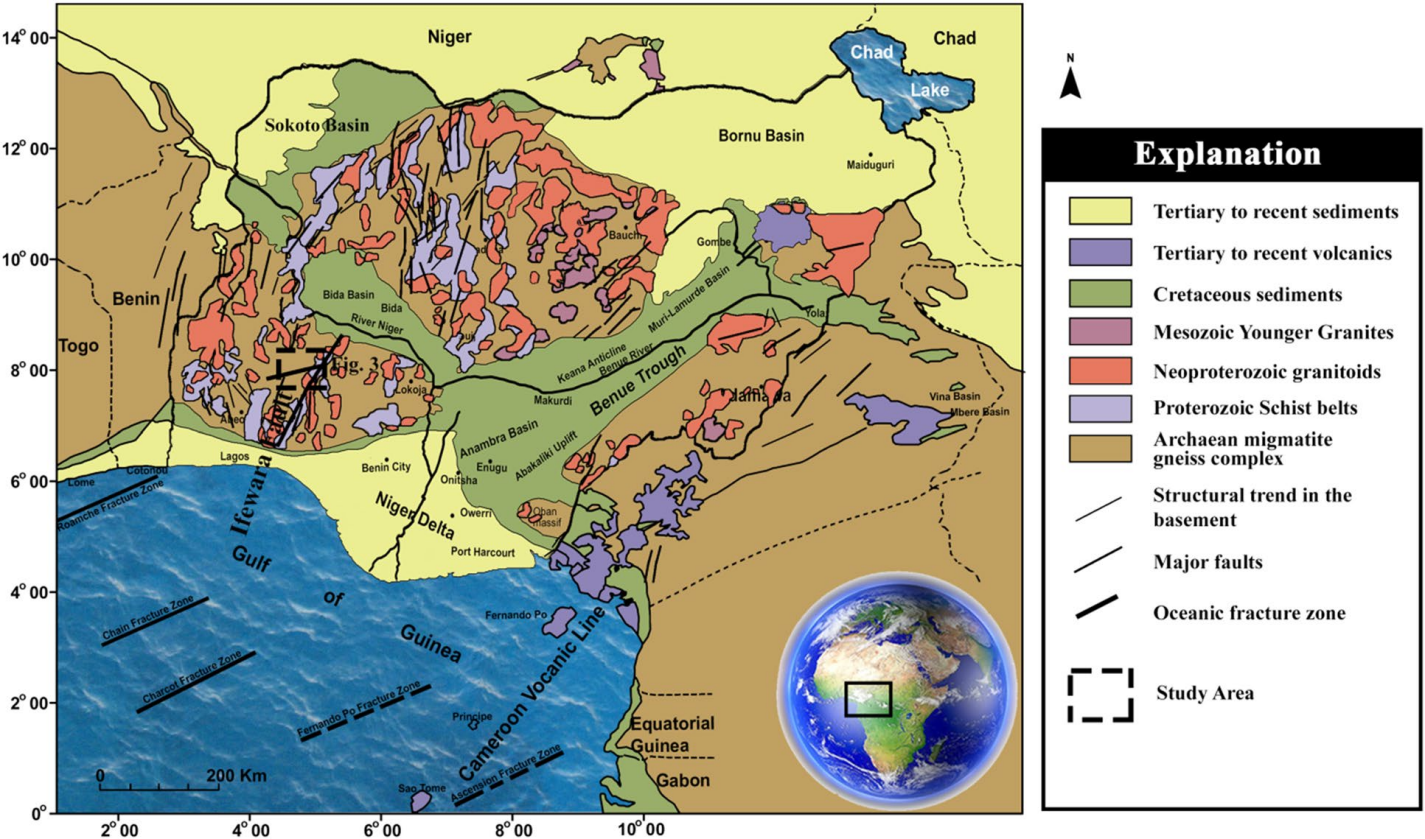
Simplified geological map of Nigeria and environ showing the study area indicated with dashed black lines (Modified after Ref.^13^).

The study area (Ife-Ilesha schist belt) falls within the basement complex of southwestern Nigeria as shown in Fig. 1. The Ife-Ilesha schist belt is characterized by monocyclic Proterozoic meta-sedimentary and meta-volcanic rock units deposited on the polycyclic Archaean basement complex rocks^10–12^.

### Archaean basement

The oldest rock units within the Ife-Ilesha schist belt are represented by the Archaean grey gneisses which crop out in southwest and northwest of the region. The rocks exhibit a monotonous major mineralogy such as amphibole, ilmenite, titanite, minor K-feldspar, quartz, plagioclase, biotite, allanite and late epidote^10,12^. Lenses of ortho-gneisses, rarely porphyritic, exhibit conserved igneous layering and occasional extremely strained enclaves, indicating that great parts of these grey gneisses originated from highly deformed and layered igneous protoliths that vary in composition from tonalite to granodiorite with slight layered trondhjemite (TTG-type). Successive high deformation has largely transformed the protoliths to banded grey gneiss. Shearing and re-folding of this tectonic-metamorphic banding were synchronous with partial melting of certain finely layered protoliths of adequate composition, generating up to 20 percent of plagioclase-rich leucosomes with small quantities of amphibole and/ or biotite, ilmenite and titanite. Anatexis of rock units of leucotonalitic to trondjhemitic composition needs regional temperatures greater than 700 °C^10,14^. Boudins and lenses of amphibolites, occasionally torn apart and cemented by plagioclase-rich leucocratic material, are similarly detected within the grey gneiss complex. Intrusive undeformed Pan-African granodiorite sheets are common. In the University of Ife campus, meta-texites are cut by un-deformed syn-metamorphic, syn-anatectic magmatic veins which ranged from granodiorite to biotite-rich tonalite and diorite, that are fairly tilted to foliation. U–Pb zircon ages of about 2.6 Ga (upper intercepts), acquired by Ref.^15^, limit the Late-Archean age of the grey gneisses from the University of Ife campus. Lower intercepts of the Concordia curve within 600 Ma affirm the main effect of high temperature Pan-African metamorphism.

### Proterozoic rock units

**Figure 2:**
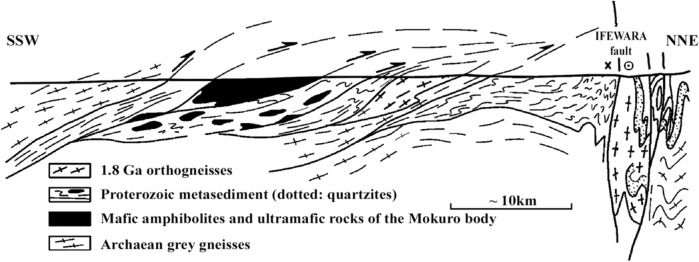
Cross-section of Ife–Ilesha schist belt within the study area, with equal vertical and horizontal scales adopted from Ref.^12^. The profile location is shown in Fig. 1. The interpretative cross-section showing geometrical relationships between Archaean basements, Proterozoic cover and the Ifewara fault which is the dominant feature of the study area as shown in Fig. 1.

In the Ife–Ilesha schist belt, monotonous sequences of meta-sediments (meta-pelites and quartz-schists) exhibit recumbent foliation and tectonically lie on the Archaean grey gneisses and pink ortho-gneisses of the Ife dome (Fig. 2). Mica-schists and quartz-schists commonly exhibit conserved sedimentary beddings which have been involved in recumbent to isoclinal folds. These schists alternate with quartzite units. Aluminous quartzites formed prominent units cropping in ridges east of the Ifewara Shear Zone, where they are associated with the pink ortho-gneisses. The mica-schists is dominated by biotite–muscovite–quartz with minor staurolite, garnet and rarely conserved fibrolite mineral assemblages^12^.

Palaeoproterozoic K-rich pink ortho-gneisses derived from porphyritic red granitoids and meta-porphyries described by Ref.^12^; represent meta-intrusive rock units emplaced in the quartzite structure. In the northwestern part of Ife, pink porphyritic ortho-gneisses form the core of a dome bounded by Archaean grey gneisses (Fig. 2). Pink augen gneisses were derived from K-rich porphyritic alkaline granite units. Iron-rich dark green biotite and amphibole of the hastingsite group represent poly-crystalline pseudo-morphs after coarse grained (greater or equal 1 cm) igneous phases. As a result of bulk mineralogy and rheology, extreme shearing is rare and very limited partial melting is observed in the orthogneisses compared to the surrounding grey gneisses. Though, late kinematic pegmatoid veins cut the foliation and display enormous amphibole and K-feldspar phenocrysts. Contacts with adjacent Archaean grey gneisses are nearly parallel to the regional foliation^10,12^. The pink sub-alkaline ortho-gneisses within the university campus and in Ibadan City together yielded an age of 1.8 Ga U–Pb zircon^15^.

### Structural styles

**Figure 3:**
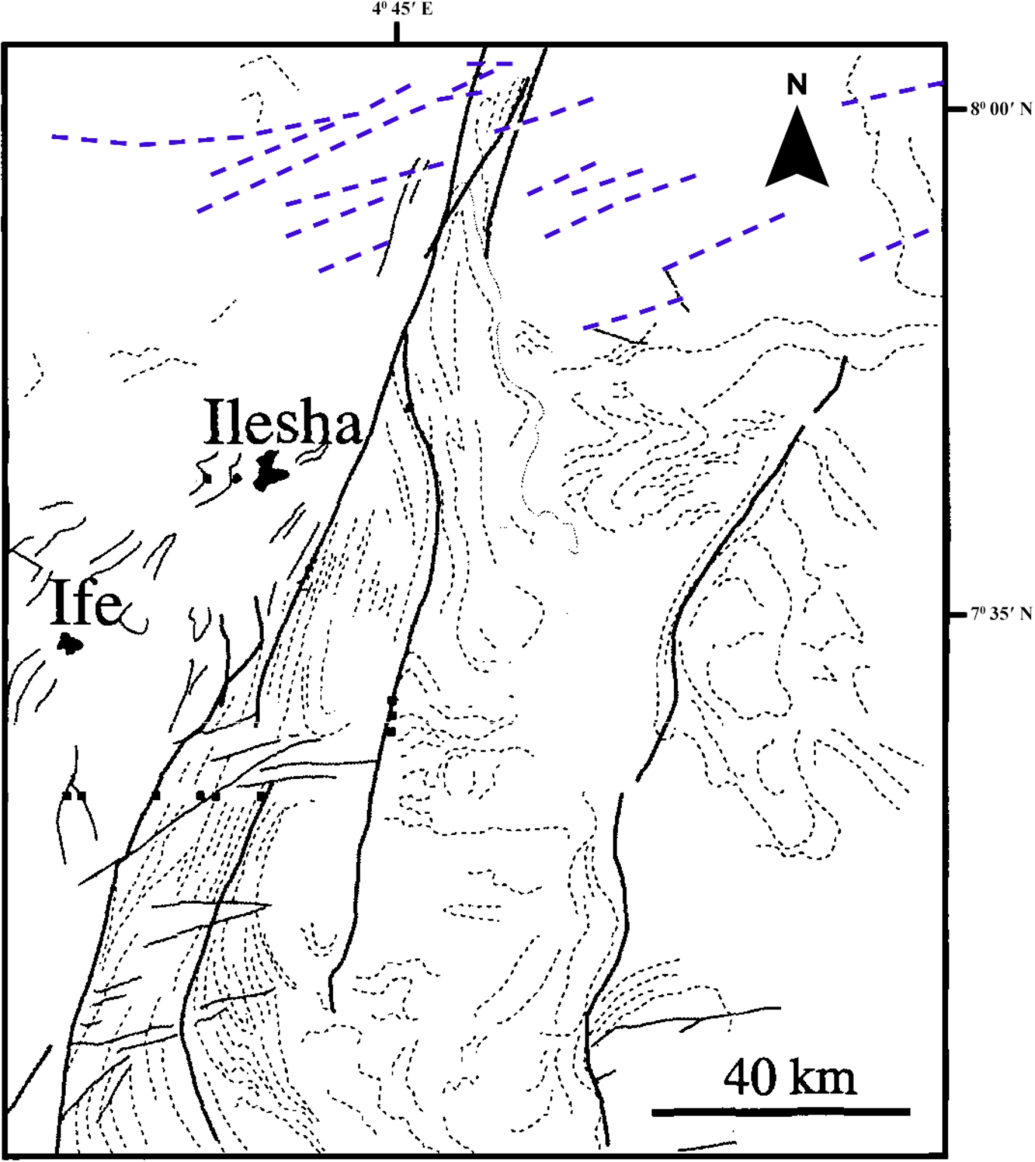
Structural map of the Ife-Ilesha schist belt established using Radar and Landsat images (modified from Ref.^10^). Dashed blue lines: NE-trending structures which host Sn–Nb–Ta–productive pegmatites Refs.^1,21^.

The structural pattern within the Ife-Ilesha schist belt is characterized by N–S vertical fold with downcast lineation, indicating a transpressive regime identical to that reported in various parts of the Tuareg shield^10,16–18^. Faults and shear zones which are characterized as lineament are concealed. Although, fractures are located on hilltops, slopes and on few outcrops of basement rocks which are exhumed along river valleys. The main tectonic structure of the Ife-Ilesha schist belt is the NNE-SSW trending Ifewara fault (Fig. 3). This structure is the southern extension of the NNE-trending Kalangai-Zungeru transcurrent fault associated with gold mineralization^19,20^. The concealed mineral deposits of the Ifewara shear zone are yet to be established. The Ifewara shear zone cross cuts the Ife-Ilesha schist belt and separates the belt into two contrasting rock assemblages.

## Data and methodology

Several techniques that comprises of magnetic, radiometric, and satellite data were utilized to establish structural features including the Ifewara fault that plays a major role in the localization of mineral deposits within the Ilesha schist belt, southwestern Nigeria.

### Satellite data

**Figure 4:**
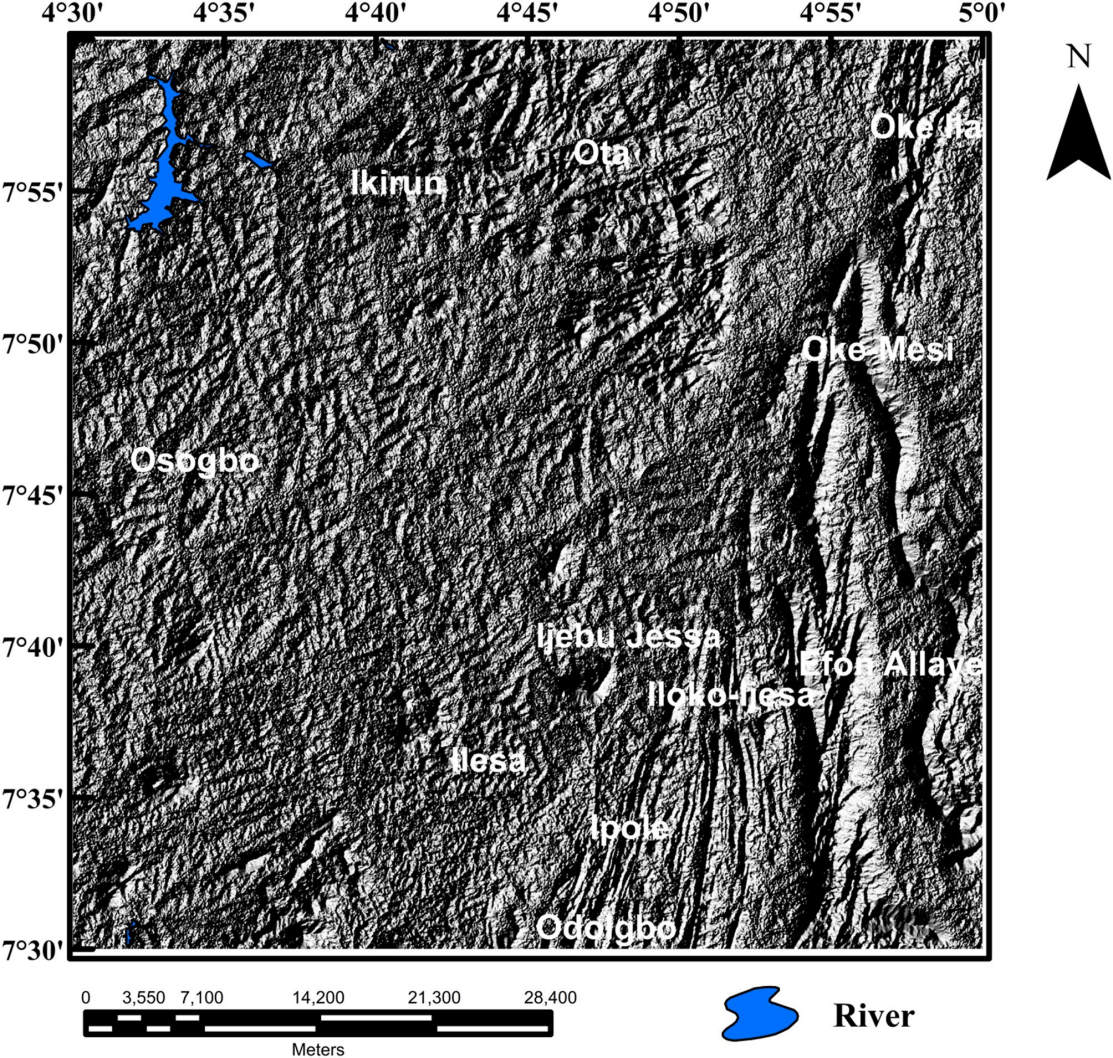
Shaded-relief map computed from Shuttle Radar Topography Mission data of Ife-Ilesha schist belt utilizing a 45 degree light angle at azimuths 112.5 degrees. Shuttle Radar Topography Mission image courtesy of the U.S. Geological Survey. The figure was produced using ArcMap Software version 10.8 (Website: https://desktop.arcgis.com/en/arcmap/).

**Figure 5:**
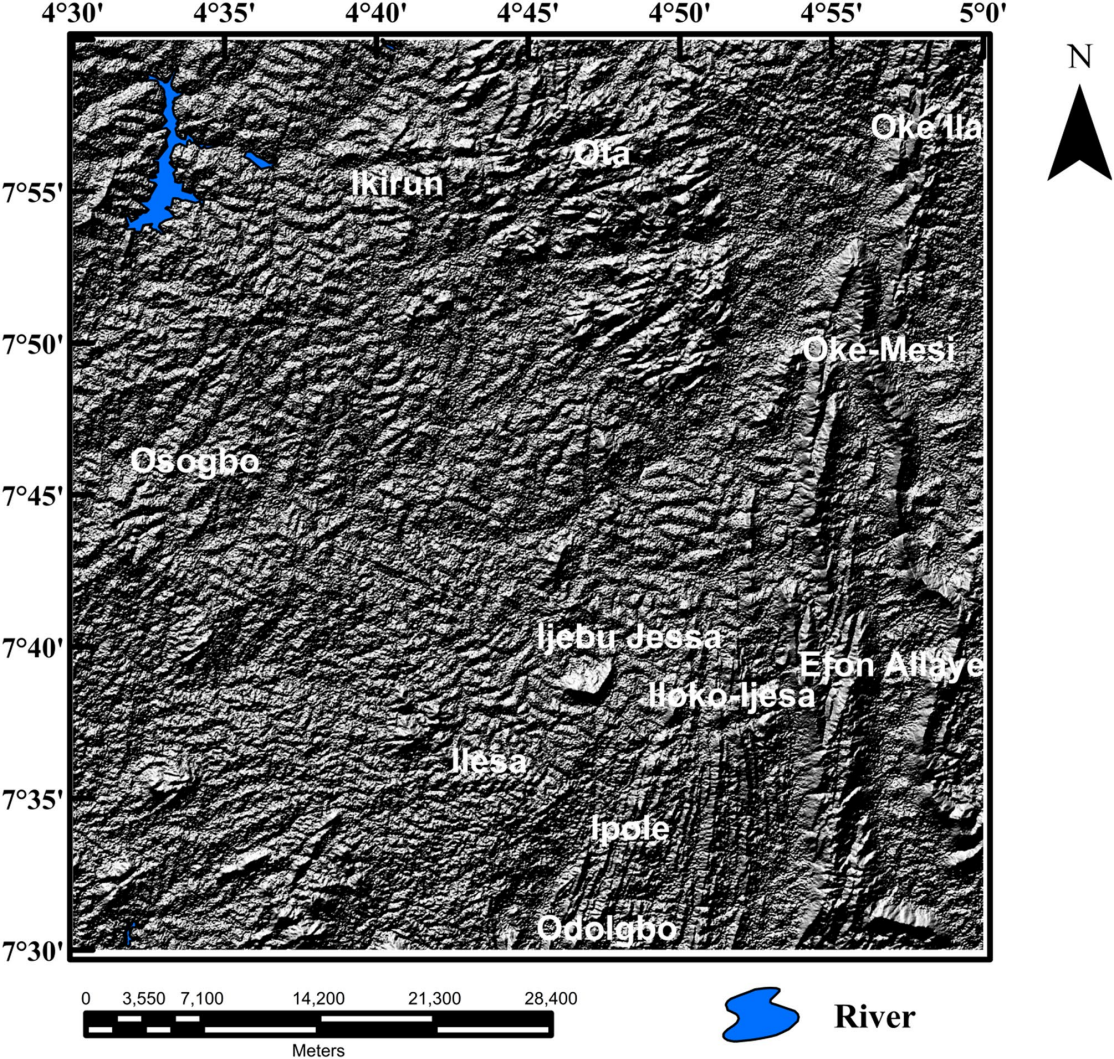
Shaded-relief map computed from Shuttle Radar Topography Mission data of Ife-Ilesha schist belt utilizing a 45 degree light angle at azimuths 337.5 degrees. Shuttle Radar Topography Mission image courtesy of the U.S. Geological Survey. The figure was produced using ArcMap Software version 10.8 (Website: https://desktop.arcgis.com/en/arcmap/).

The Shuttle Radar Topographic Mission (SRTM) digital elevation data of the Ife-Ilesha schist belt was downloaded from the website of the United State Geological Survey (USGS). The spatial resolution of the SRTM data is 3 arc seconds (approximately 90 m). The SRTM digital elevation data of the study area was hill-shaded using 45° light angle at azimuths of 112.5° and 337.5° (Figs. 4, 5) to highlight east-northeast and north-northeast structural trends, respectively; which are the main regional structural trends. The produced shaded-relief maps (Figs. 4, 5) will help reveal the influence of the subsurface structural features structures on the topography of the studied region. The Line algorithm of PCI Geomatica software was used to extract different lengths and directions of morphological features on the shaded-relief maps, on the basis of variation in surface features using the values of the six parameters (Table 1). Finally, rose diagram was produced from lineaments extracted from the shaded-relief maps to assess trends of surface structural features within the region.

**Table 1:** The used parameters for the automatic extraction of lineaments from the SRTM data of the study area.

Parameters	RADI	GTHR	LTHR	FTHR	ATHR	DTHR
Values	30	100	30	20	1	2

*RADI* filter radius, *GTHR* edge gradient threshold, *LTHR* curve length threshold, *FTHR* line fitting error threshold, *ATHR* angular difference threshold, *DTHR* linking distance threshold.

### Aeromagnetic and radiometric data

The total field anomaly and airborne radiometric data investigated were derived from the regional aeromagnetic and radiometric datasets of Nigeria. The datasets were acquired by a geophysical company (Fugro Airborne Surveys) on behalf of the Nigerian Geological Survey Agency between 2004 and 2009. The datasets were collected at an interval of 1.0 s (approximately 75 m) for radiometric and 0.1 s (approximately 7.0 m) for aeromagnetic measurements. The aeromagnetic survey was recorded at a sensor mean terrain clearance of 80 m on a series of NW–SE oriented flight lines (perpendicular to regional trends). The flight lines were spaced 500 and tied at 2000 m. The aeromagnetic data was corrected for diurnal variations with the main component of the geomagnetic field (IGRF) also removed.

The processing routine of the resulting total field anomaly (TFA) and radiometric data comprised of the following phases: (a) Application of total gradient amplitude (TGA) technique to the TFA data to produce the TGA map of the region which reveals structural features; (b) extraction of maxima of the TGA to produce magnetic structural map; (c) production of rose diagram from the maxima of TGA to established trends of lineaments; (d) application of 3-D Euler deconvolution technique to aeromagnetic anomaly data to estimate possible depths of structural features; and (e) production of radiometric RGB ternary map to reveal the radiometric signature of the magnetic lineaments. All color shaded relief maps of the aeromagnetic and radiometric data have been generated with an illumination inclination of 45 degrees and declination of 45 degrees.

## Results

In order to map structural features that could host mineral deposits within the study area, four steps were followed. In the first step, maxima of the total gradient amplitude (TGA) were extracted to produce the structural map of the studied region. In the second step, the extracted lineaments from the maxima of the TGA map were superimposed on the 3-D Euler deconvolution map. This is to correlate TGA lineaments with the 3-D Euler deconvolution depth map, which gives an indication of the depth to the top of the lineaments in the basement. The third step represents the superposition of the TGA lineaments on the ternary map of the study area to reveal valuable information for the identification of structural features associated with economic mineral deposits. Finally, in the fourth step, morphological lineaments extracted from the shaded relief map were compared with structural features extracted from the TGA map. This is to spatially correlate how subsurface lineaments in the basement influence morphological features of the region.

### Morphological lineaments

The shaded-relief maps of the study area are produced using the Shuttle Radar Topographic Mission elevation data. The topography features of the region are clearly depicted on the map (Figs. 4, 5). The map reveals topographical features which are controlled by subsurface structures and hence allow the automatic extraction of morphological features. In order to ensure quality interpretations and identification of extension of subsurface lineaments, morphological lineaments were used in combination with magnetic subsurface structures.

### Magnetic lineaments

**Figure 6:**
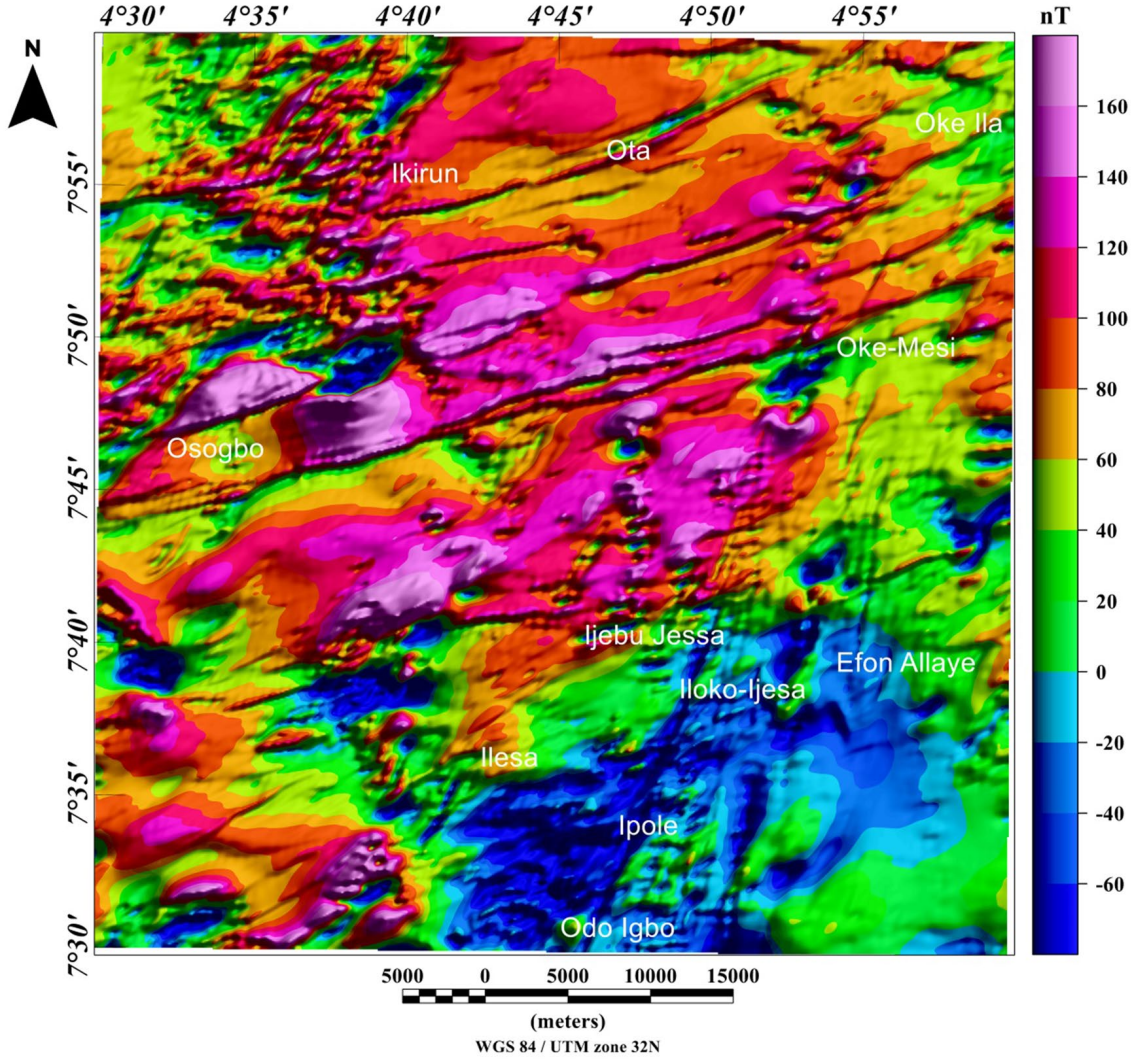
Color-shaded total field anomaly map of Ife-Ilesha belt showing large variations in the magnitude of aeromagnetic anomaly dataset and color shading highlighting linear structures. The NNE–trending magnetic anomalies extending from the southern part of the map through Odo Igbo to Iloko-Ijesa, similar to Ifewara fault zone shown on the geological map (Fig. 1) of the study area. In addition, for low-latitude areas such as the study region amplitudes of anomalies resulting from magnetic sources changes from high to low values. The figure was produced using Oasis Montaj Software version 8.3.3 (Website: https://www.seequent.com/).

**Figure 7:**
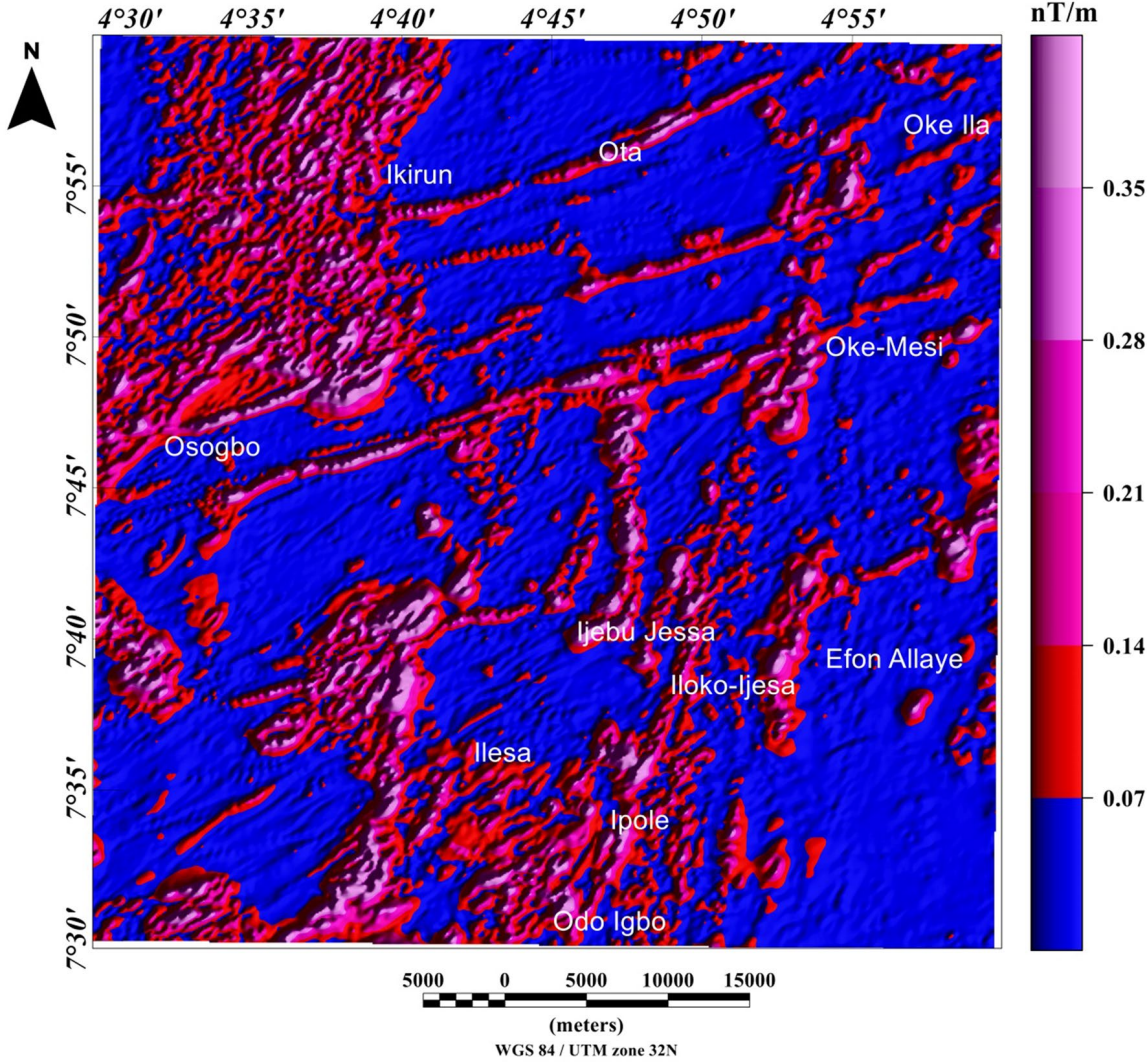
Total gradient amplitude map produced from the total field anomaly data of the study area presented in color-shaded relief, illuminated from the east-southeast direction. The east-southeast illumination (azimuth: 112.5 degrees and light angle: 45 degrees) enhances east-northeast lineaments. The figure was produced using Oasis Montaj Software version 8.3.3 (Website: https://www.seequent.com/).

**Figure 8:**
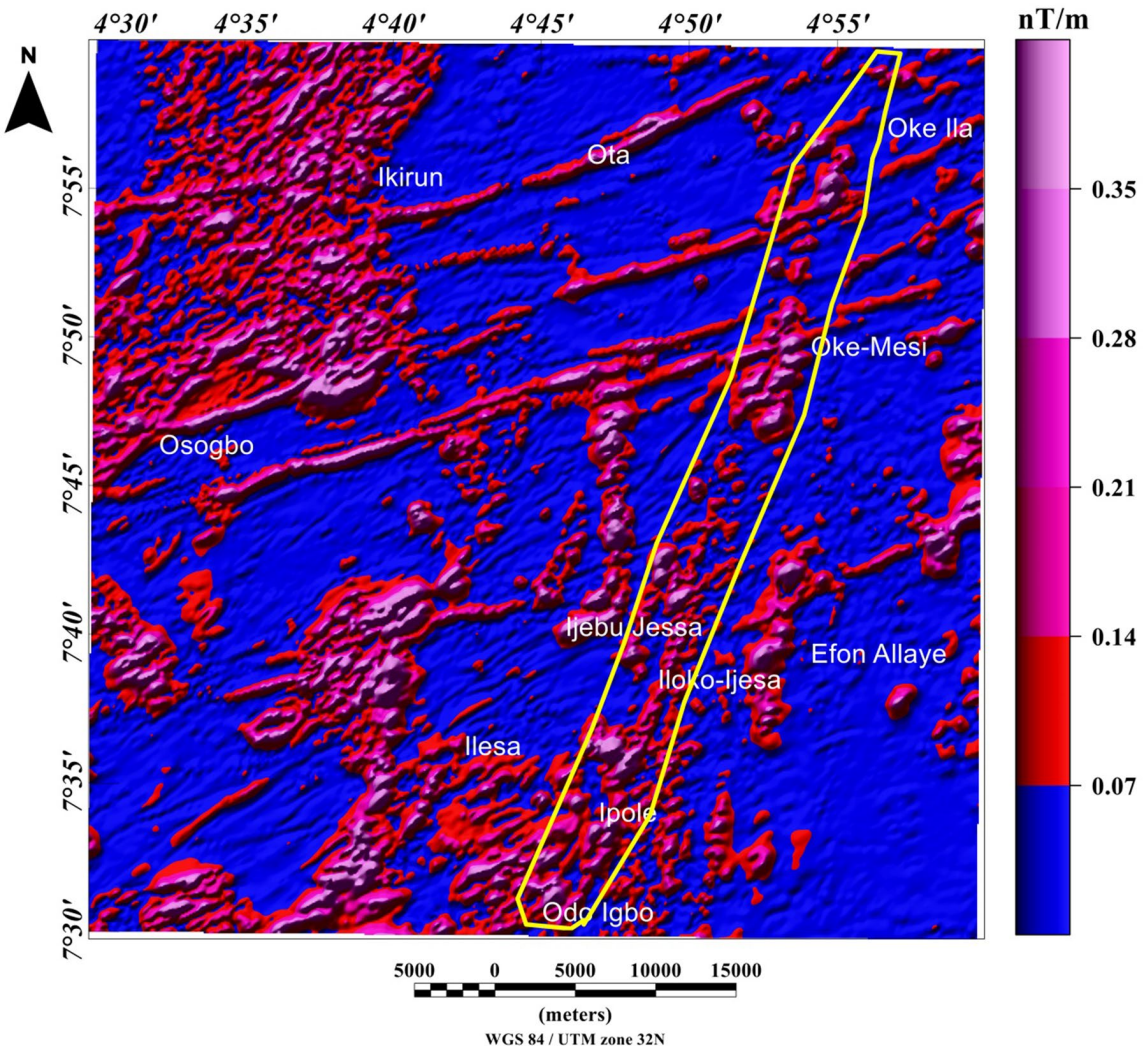
Total gradient amplitude (TGA) map produced from the total field anomaly data of the study area presented in color-shaded relief, illuminated from the north-northwest direction. The north-northwest illumination (azimuth: 337.5 degrees and light angle: 45 degrees) enhances north-northeast lineaments. The color shading highlights the NNE-trending Ifewara fault which is revealed in areas represented within the yellow polygon on TGA map. The figure was produced using Oasis Montaj Software version 8.3.3 (Website: https://www.seequent.com/).

**Figure 9:**
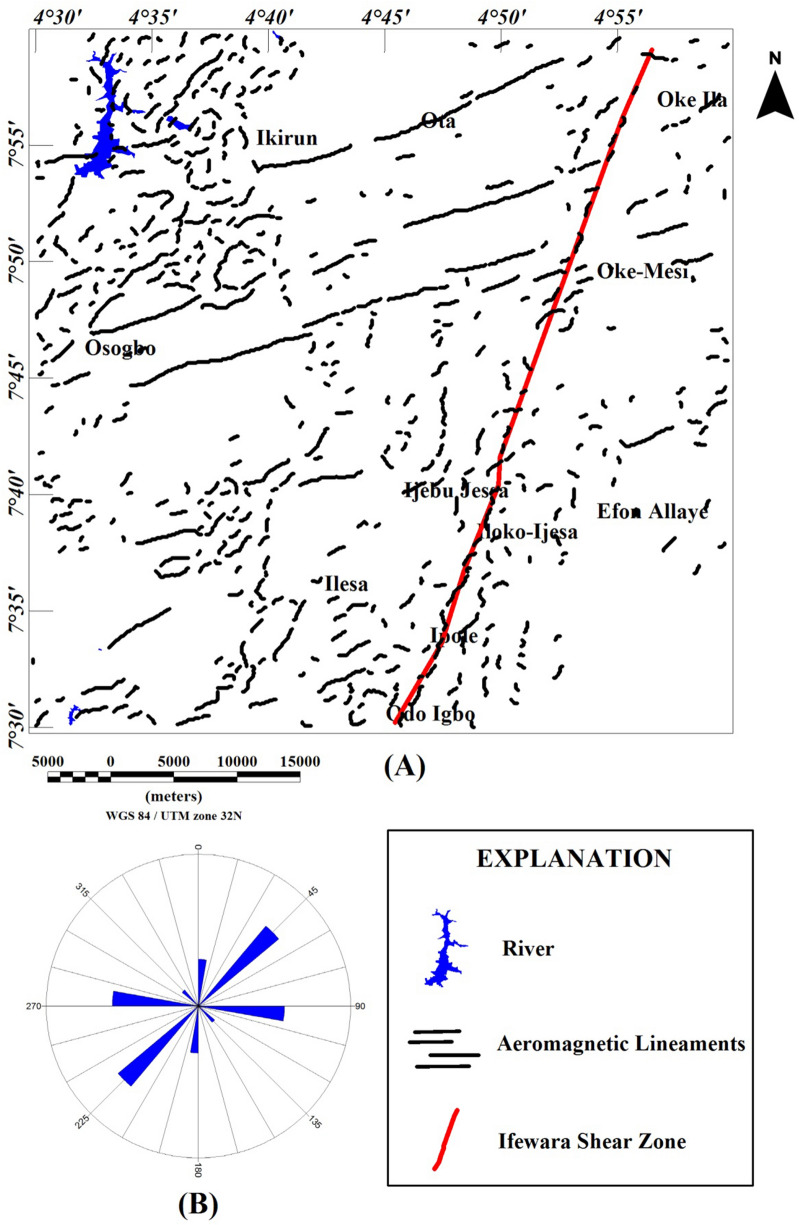
(**A**) Magnetic structural map showing extracted maxima of the total gradient amplitude (TGA), which was used for locations and lateral extent of structural features. (**B**) Rose diagram produced from the maxima of TGA, showing major NE-SW and E-W structural trends. (**A**) was produced using Oasis Montaj Software version 8.3.3 (Website: https://www.seequent.com/).

The visual inspection of the total field anomaly (TFA) map (Fig. 6), shows high (green to magenta colors) and low (deep and light blue colors) anomalies values between − 60 and 160 nT. The TFA map reveals magnetic high values, which are located at the central and northerly parts of the study area, portrayed by red and magenta colors with amplitude values between 60 and 160 nT. The magnetic high value anomalies are trending mainly in the ENE-WSW directions. Magnetic low values are located mainly in the southwest and few at the central part of the studied region, portrayed by deep blue colors with amplitude values between − 60 and − 20 nT. The magnetic low value anomalies are trending NNE-SSW directions. The TFA map reveals subtle linear magnetic anomalies within different parts of the study area. The utilization of TFA lineaments for the identification of structurally controlled mineralization is inherently subjective, particularly with respect to possible remanent magnetization in the TFA data and also due to polarity effects. In some locations it is possible that dipolar anomaly features may be mistaken for anomalies that are directly centered above their sources. As such, the TFA data requires enhancement filter such as total gradient amplitude (TGA) which is independent of remanent magnetization to confirm the nature of TFA anomalies. It should be noted that the TGA technique is independent of remanent magnetization for 2-D magnetic structures^22^. However, the TGA technique is not independent of the directions of source magnetization and ambient magnetic field for the 3-D magnetic sources^22,23^. The total gradient amplitude (TGA) map produced from the total field anomaly data enhanced the magnetic signature of geologic structures within the study area. The TGA maps are presented in Figs. 7 and 8 as color shaded-relief maps with two illumination directions. This is to enhance the signature of the WSW-ENE and NNE-SSW trending structural features (such as fractures, faults and intrusions) that dominate the TFA map (Fig. 6). Magnetization contrasts within the study area which are indicated by maxima of the TGA are emphasized on Figs. 7 and 8. In particular, a major structural feature can be seen in Fig. 8 (indicated with yellow polygon) that has been identified as the Ifewara shear zone. The maxima of the TGA map were extracted automatically to produce the structural map (Fig. 9A) of the region. This is to highlight the lateral extent and locations structural features within the studied region. In addition, we produced the Rose diagram (Fig. 9B) of these structural features, which reveal two minor and major trends, with the former trending N–S and NW–SE directions and the later trending NE–SW and E–W directions. The TGA lineaments can be associated to faults and intrusions that generate simple TGA maxima, as the case may be for structures that cross various types of terrain and lithologies within the study area.

### Spatial correlation of lineaments and depth estimation

**Figure 10:**
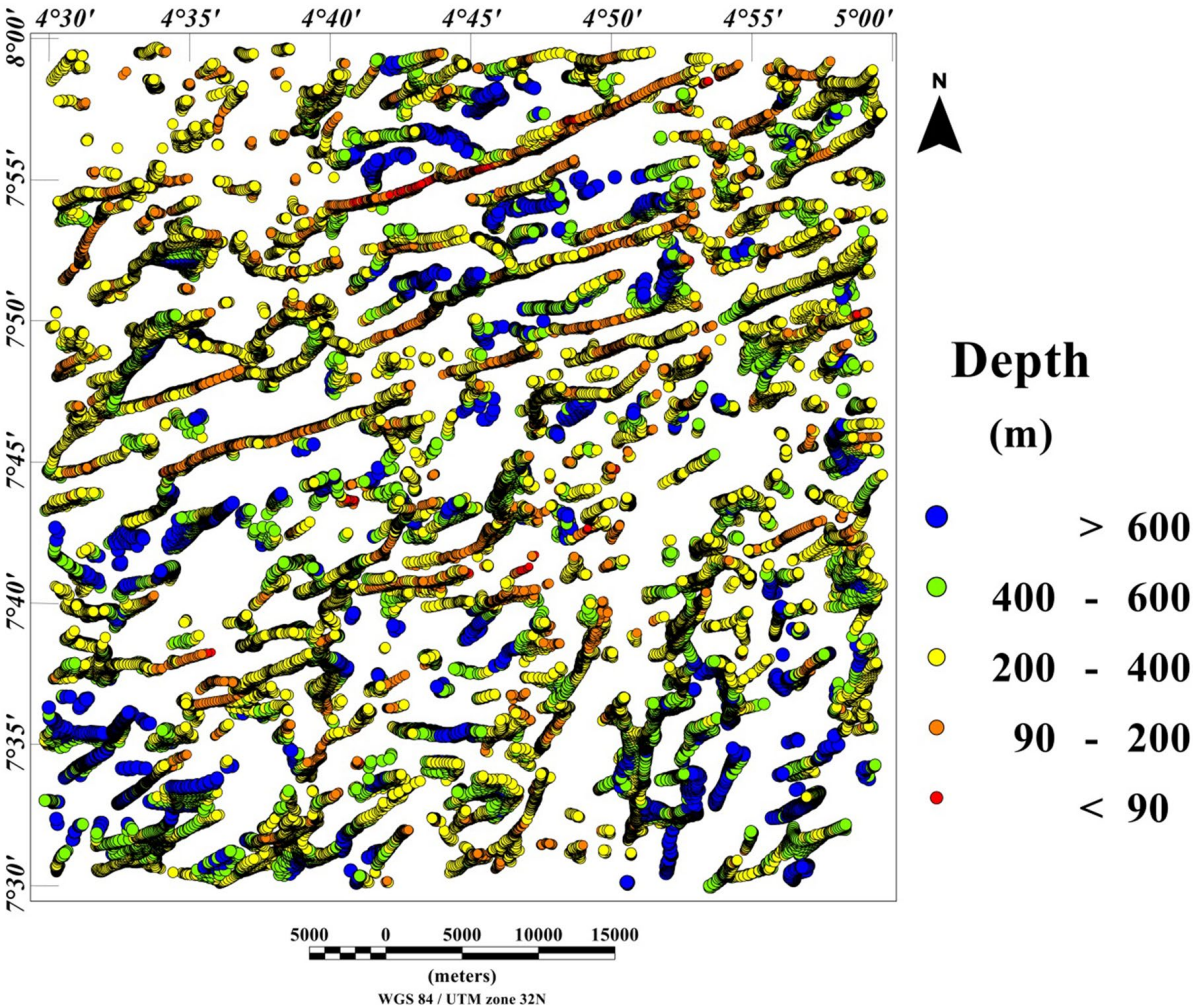
3-D Euler deconvolution depth solution map generated from the total field anomaly data using SI = 1 for structural features of the Ife-Ilesha schist belt. The figure was produced using Oasis Montaj Software version 8.3.3 (Website: https://www.seequent.com/).

**Figure 11:**
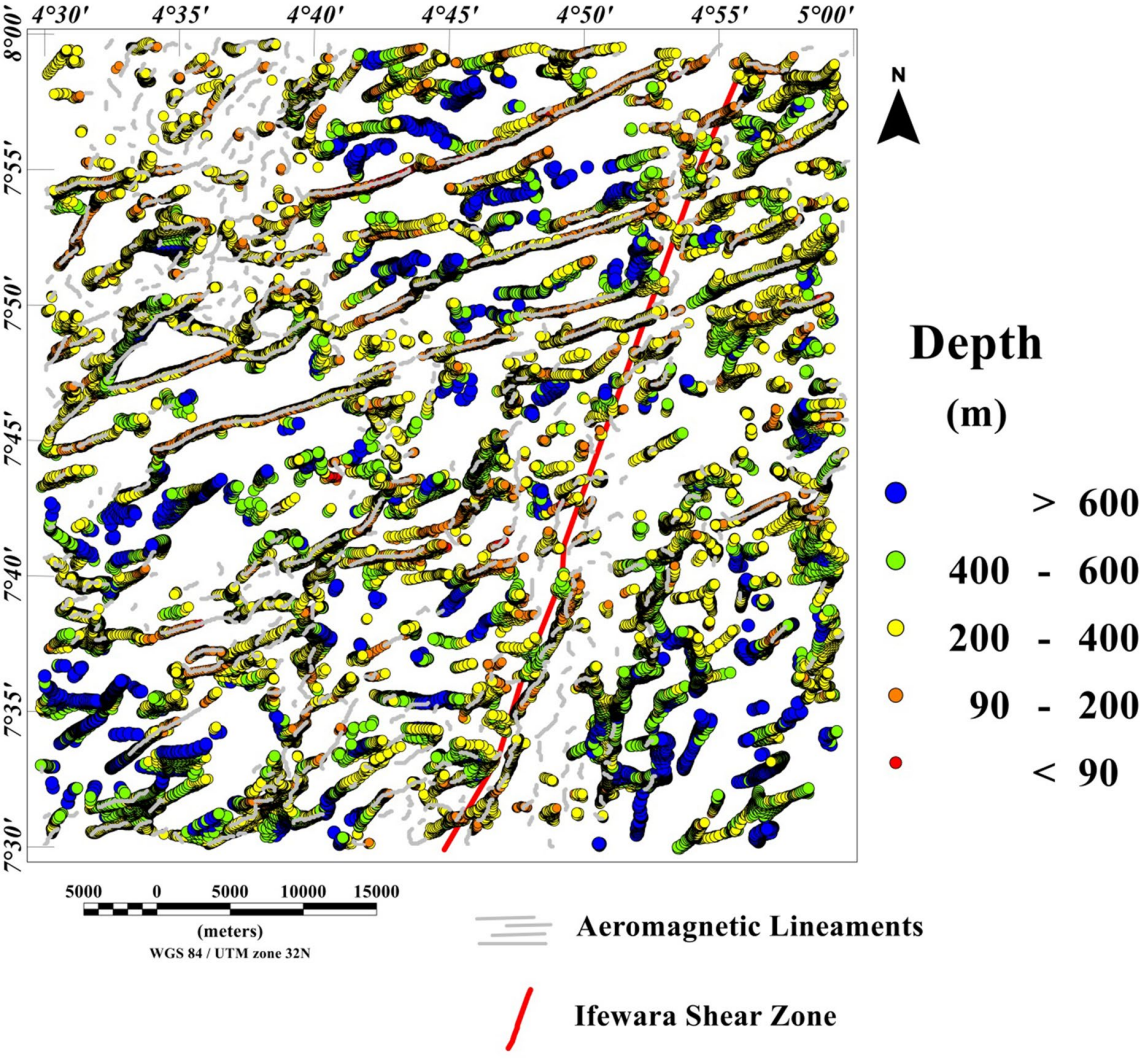
3-D Euler deconvolution map with superimposed lineaments delineated from TGA method, revealing good correlation of TGA lineaments with 3-D Euler depth solutions.

The 3-D Euler deconvolution technique has been applied on the total field anomaly data utilizing a window size of 2000 m width and a maximum depth tolerance of 8%. In addition, we utilized a structural index of one, to estimate possible depth values of the lineaments extracted from the maxima of the total gradient amplitude (TGA) shown in Fig. 9A. The TGA lineaments were superimposed on the 3-D Euler deconvolution map (Fig. 10), for good understanding of lineaments origin as shown in Fig. 11. Very good agreement was observed between the 3-D Euler depth solutions and TGA lineaments in Fig. 13. It was observed that depth values of the lineaments extracted from the maxima of the TGA map varies from 90 to 200 m with an average value of 145 m. Notable, the magnetic sources within the Ifewara shear zone which has surface manifestation as revealed by the shaded-relief maps (Figs. 4, 5), is well evident on the 3-D Euler deconvolution map with a minimum depth value of about 90 m to the top of the sources within the shear zone. This indicates that the magnetic lineaments within the Ife-Ilesha schist belt occur at shallow depth which can be accessed by miners for exploitation of rich mineral deposits.

### Spatial correlation of aeromagnetic lineaments with radiometric anomalies

**Figure 12:**
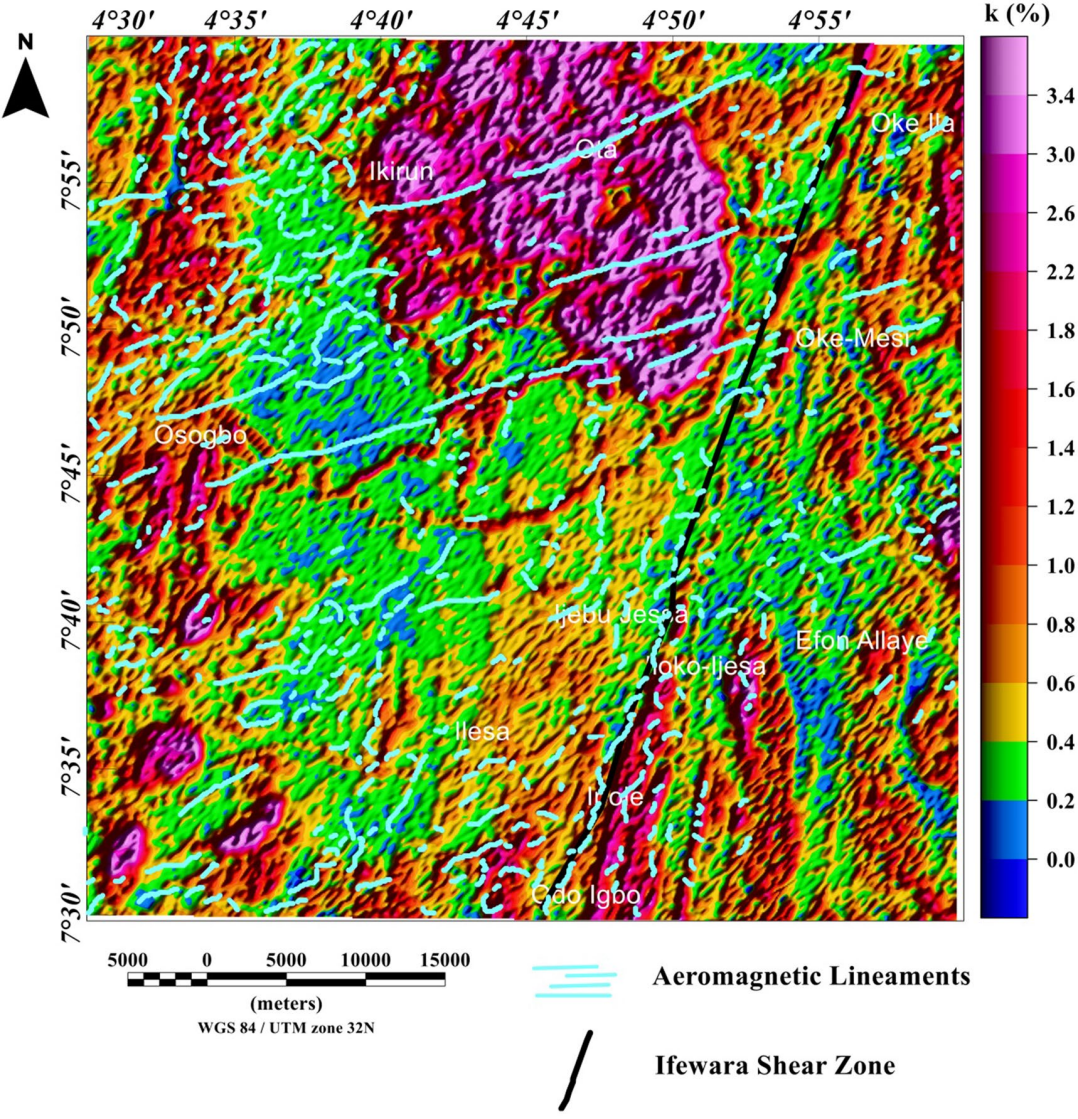
Map of potassium (%) data of the Ife-Ilesha schist belt with superimposed Ifewara shear zone extracted from the total gradient amplitude map presented in color-shaded relief, illuminated from the east-southeast direction. The east-southeast illumination (azimuth: 112.5 degrees and light angle: 45 degrees) enhances east-northeast lineaments. The figure was produced using Oasis Montaj Software version 8.3.3 (Website: https://www.seequent.com/).

**Figure 13:**
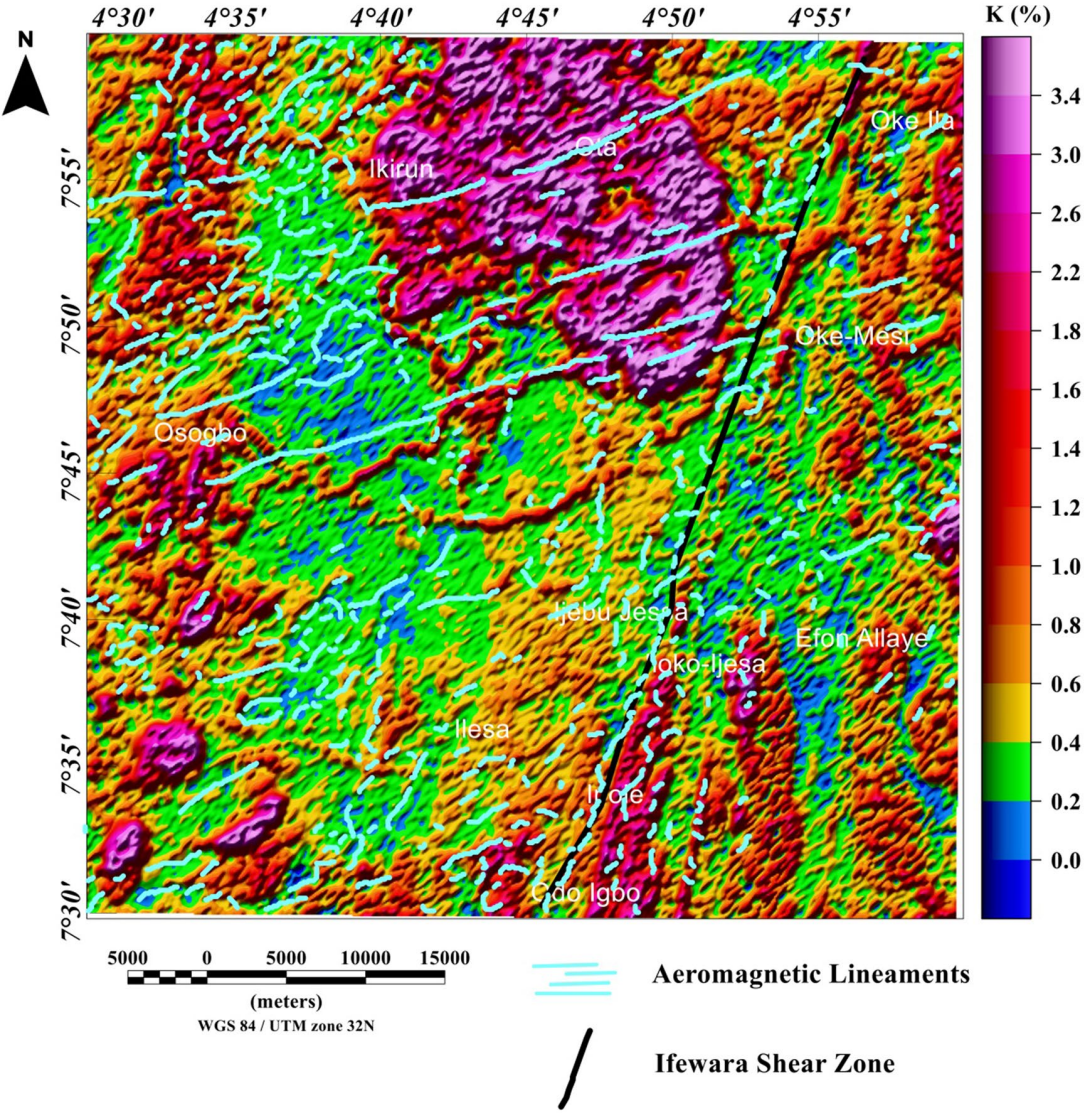
Map of potassium (%) data of the Ife-Ilesha schist belt with superimposed Ifewara shear zone extracted from the total gradient amplitude map presented in color-shaded relief, illuminated from the north-northwest direction. The north-northwest illumination (azimuth: 337.5 degrees and light angle: 45 degrees) enhances north-northeast lineaments. The figure was produced using Oasis Montaj Software version 8.3.3 (Website: https://www.seequent.com/).

**Figure 14:**
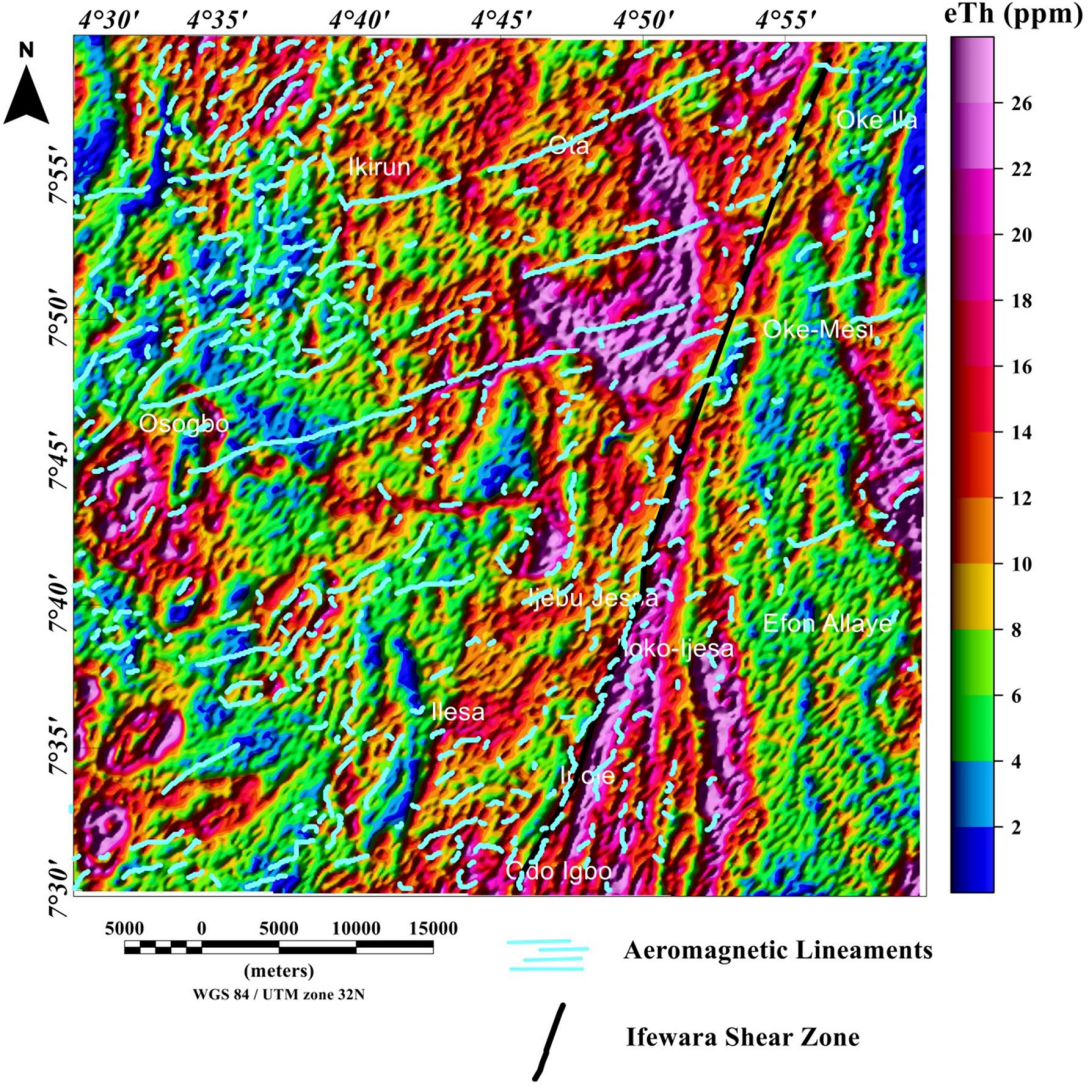
Map of thorium (ppm) data of the Ife-Ilesha schist belt with superimposed Ifewara fault zone extracted from the total gradient amplitude map presented in color-shaded relief, illuminated from the east-southeast direction. The east-southeast illumination (azimuth: 112.5 degrees and light angle: 45 degrees) enhances east-northeast lineaments. The figure was produced using Oasis Montaj Software version 8.3.3 (Website: https://www.seequent.com/).

**Figure 15:**
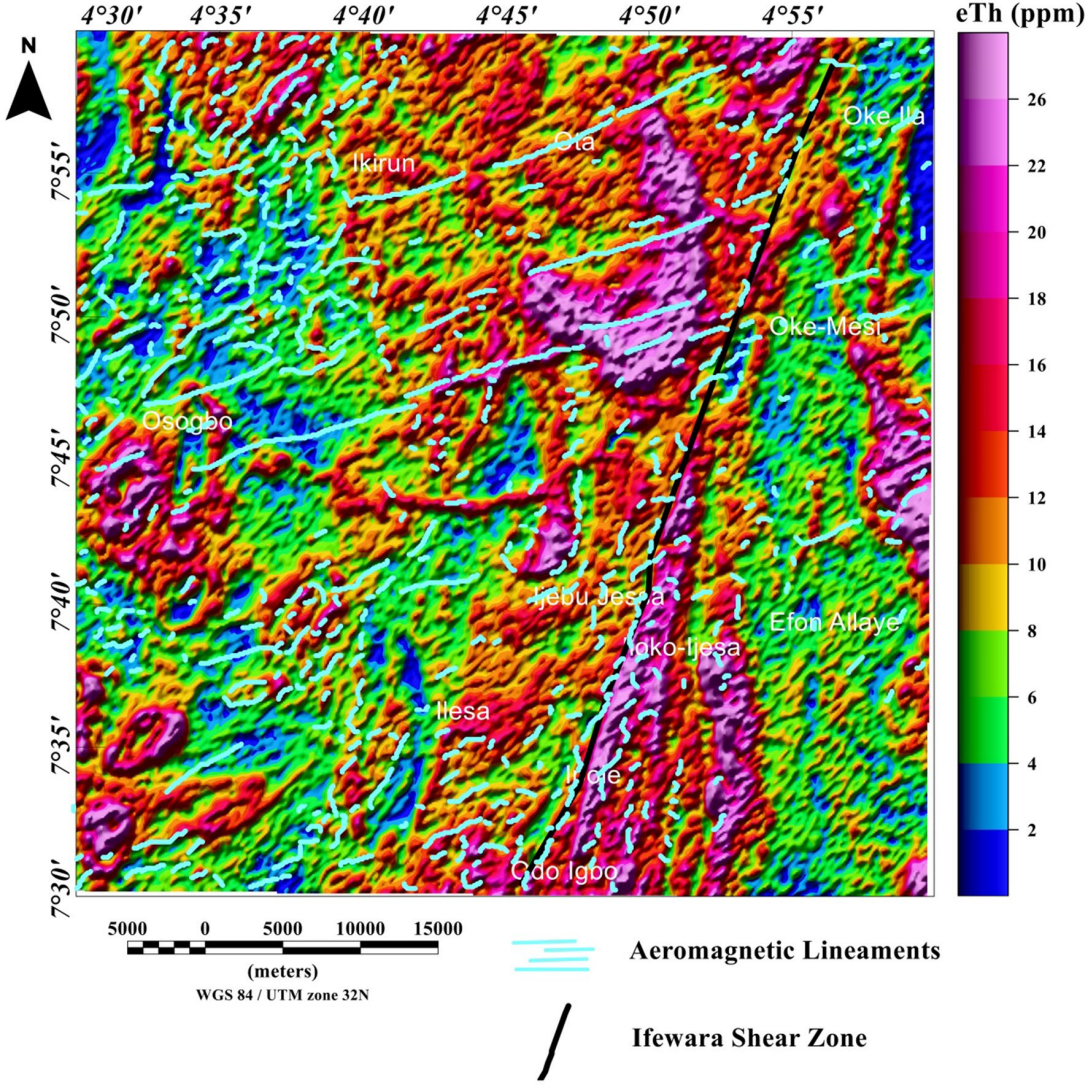
Map of thorium (ppm) data of the Ife-Ilesha schist belt with superimposed Ifewara fault zone extracted from the total gradient amplitude map presented in color-shaded relief, illuminated from the north-northwest direction. The north-northwest illumination (azimuth: 337.5 degrees and light angle: 45 degrees) enhances north-northeast lineaments. The figure was produced using Oasis Montaj Software version 8.3.3 (Website: https://www.seequent.com/).

**Figure 16:**
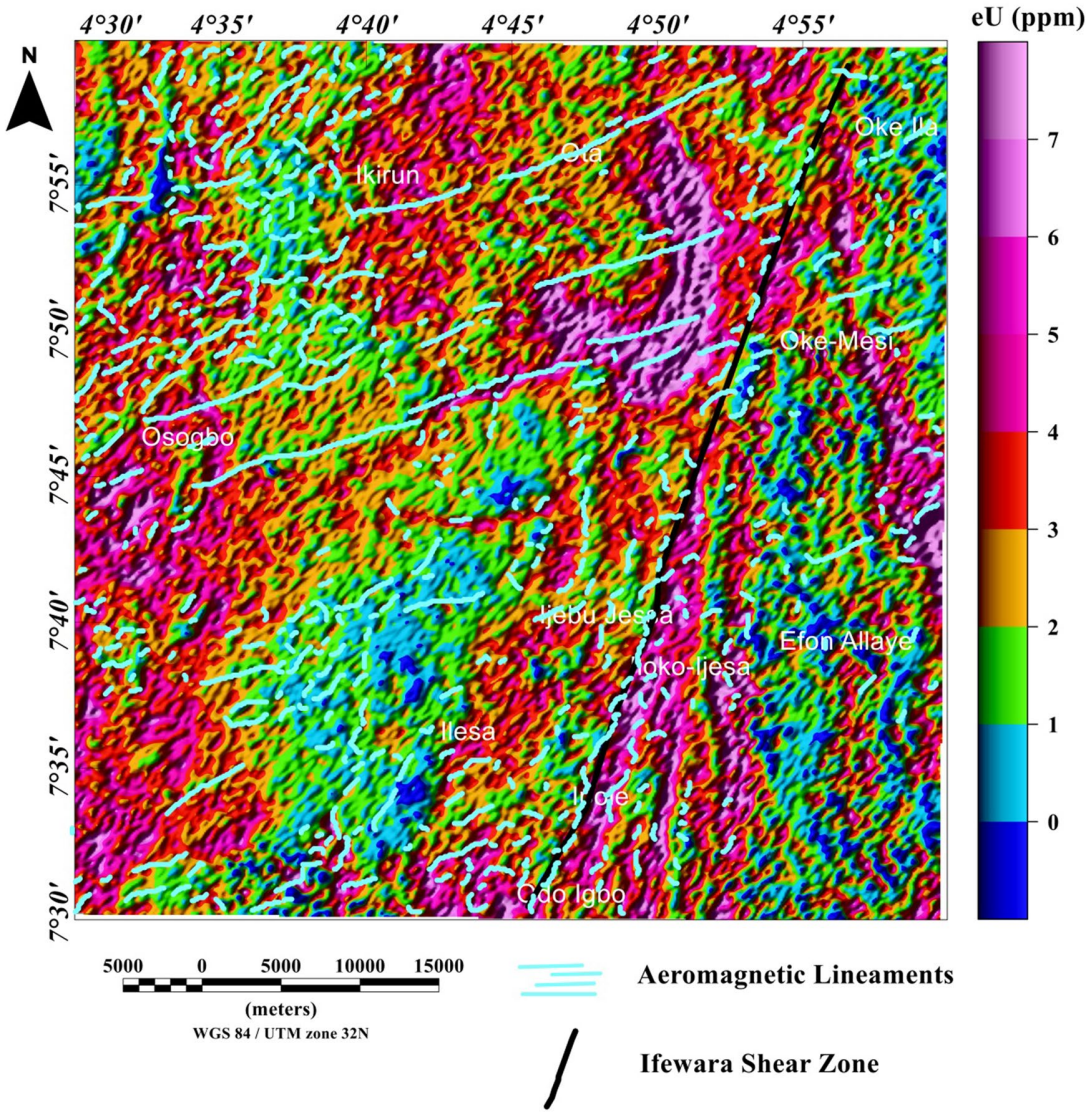
Map of uranium (ppm) data of the Ife-Ilesha schist belt with superimposed Ifewara fault zone extracted from the total gradient amplitude map presented in color-shaded relief, illuminated from the east-southeast direction. The east-southeast illumination (azimuth: 112.5 degrees and light angle: 45 degrees) enhances east-northeast lineaments. The figure was produced using Oasis Montaj Software version 8.3.3 (Website: https://www.seequent.com/).

**Figure 17:**
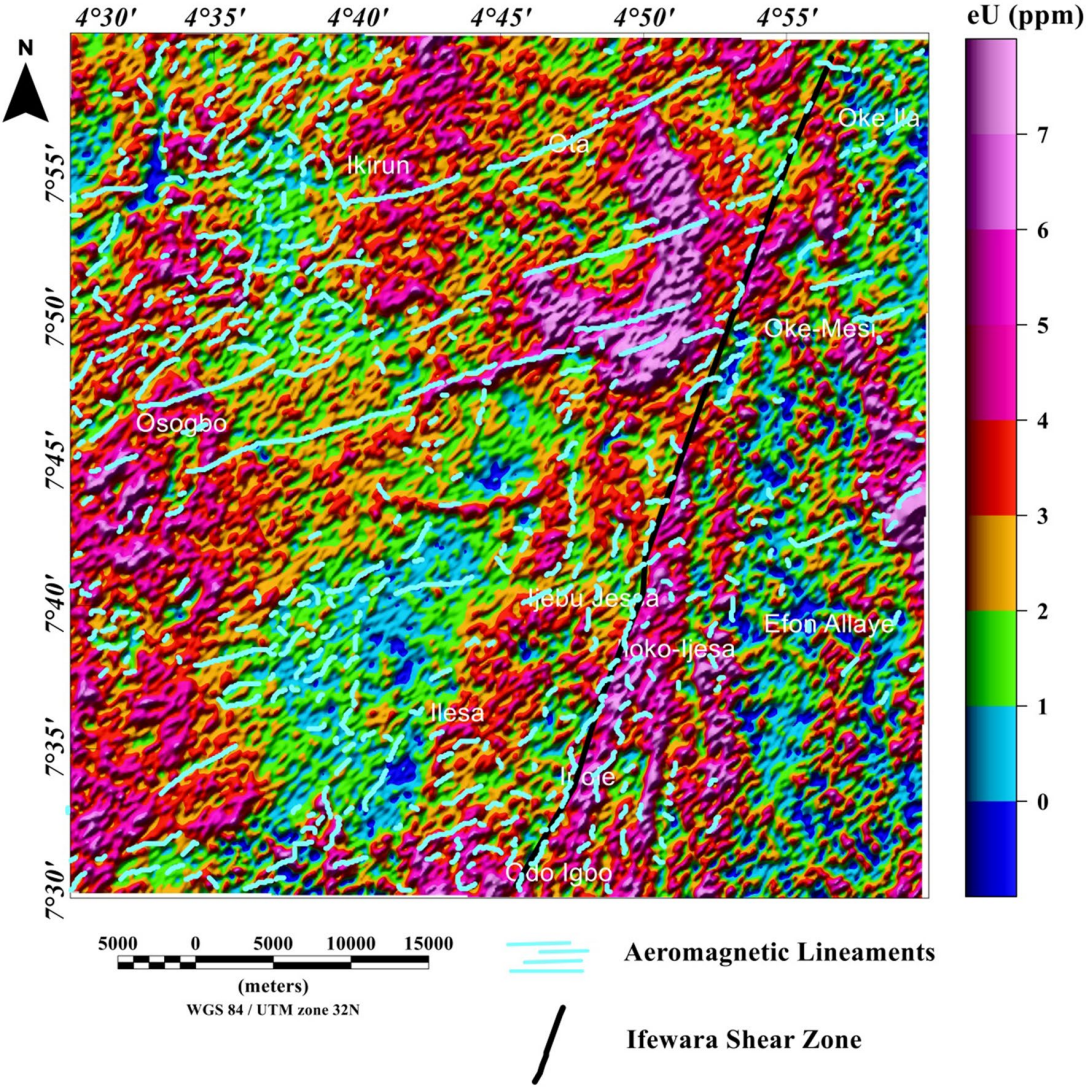
Map of uranium (ppm) data of the Ife-Ilesha schist belt with superimposed Ifewara fault zone extracted from the total gradient amplitude map presented in color-shaded relief, illuminated from the north-northwest direction. The north-northwest illumination (azimuth: 337.5 degrees and light angle: 45 degrees) enhances north-northeast lineaments. The figure was produced using Oasis Montaj Software version 8.3.3 (Website: https://www.seequent.com/).

**Figure 18:**
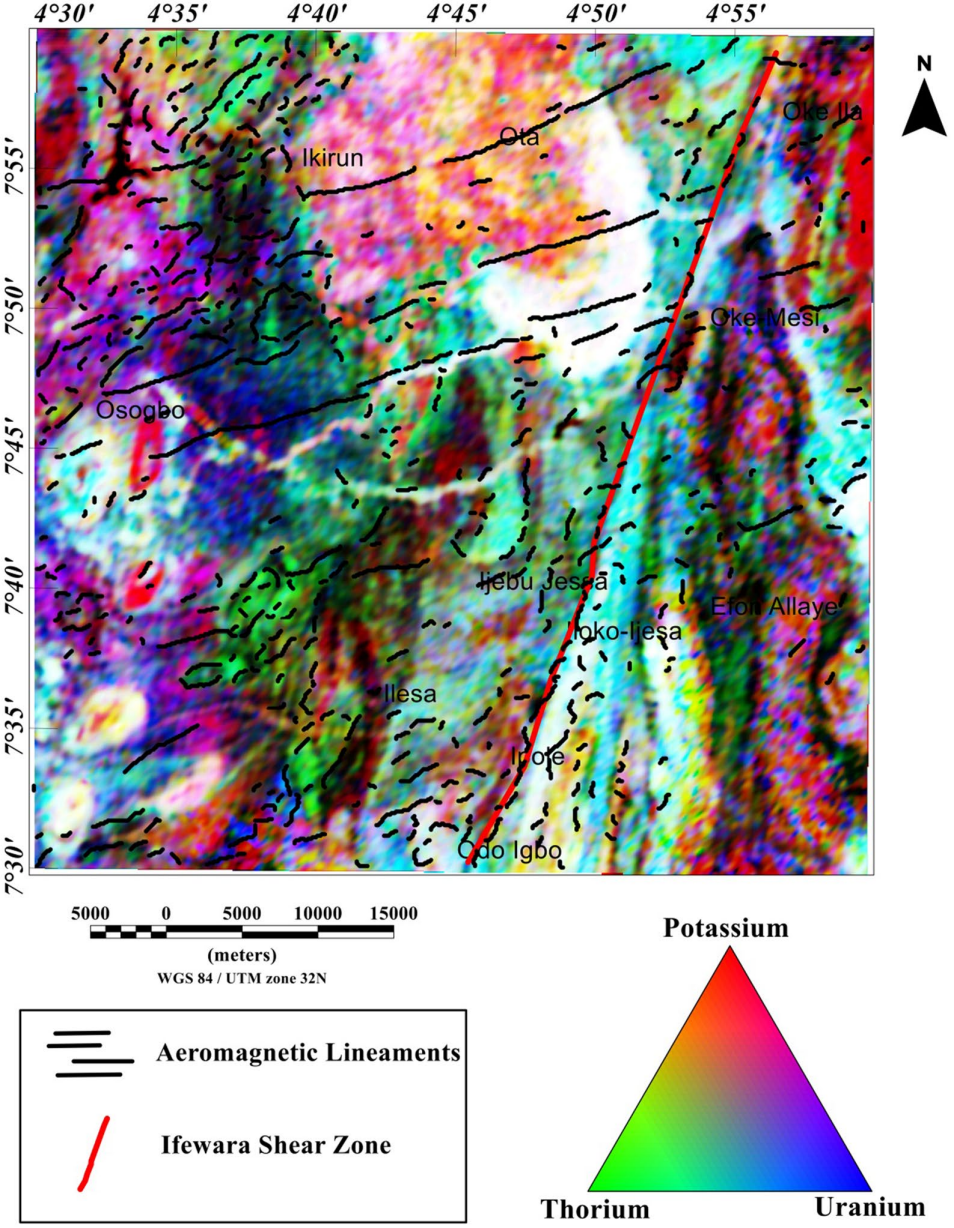
Ternary map of the Ife-Ilesha schist belt produced from the radiometric data of the region. The black and red lines show the aeromagnetic lineaments and Ifewara shear zone respectively, extracted from the total gradient amplitude map. The figure was produced using Oasis Montaj Software version 8.3.3 (Website: https://www.seequent.com/).

**Figure 19:**
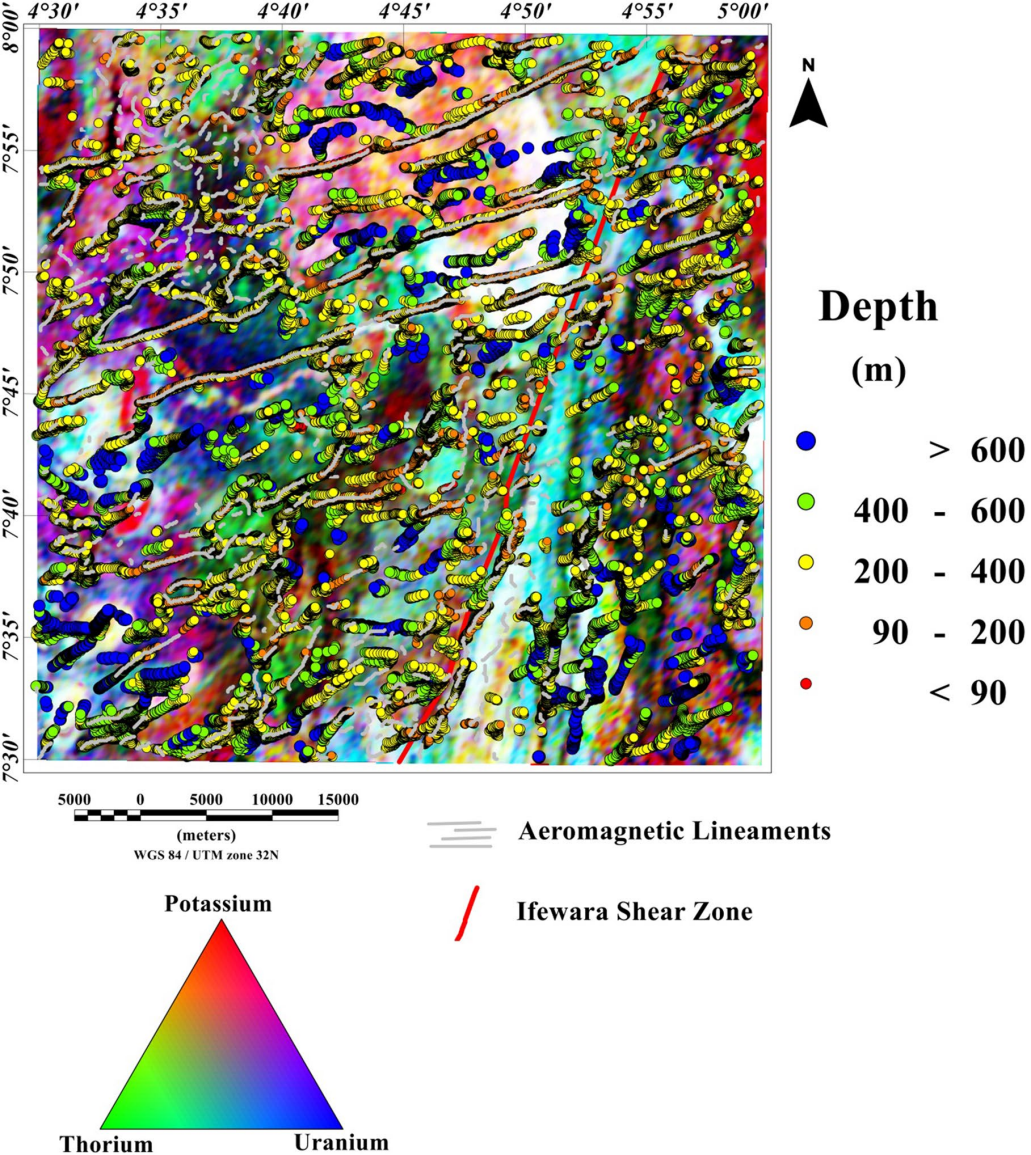
Ternary map of the Ife-Ilesha schist belt with superimposed 3-D Euler depth solutions and aeromagnetic lineaments.

The potassium, thorium, and uranium grids are displayed in Figs. 12, 13, 14, 15, 16 and 17 as color-shaded relief maps with two illuminations (45° light angle at azimuths of 112.5° and 337.5°) directions. The detected aeromagnetic lineaments and Ifewara shear zone was superimposed on the shaded-relief potassium, thorium, and uranium maps (Figs. 12, 13, 14, 15, 16, 17, respectively) to relate observed radiometric concentration with the shear zone. The Ifewara shear zone is characterized by transition between high and low radiometric anomalies, indicating major shear zone, which juxtapose large volumes of various rock units at depth. The potassium, thorium, and uranium data were combined to produce the ternary radiometric map (Fig. 18) of the study area. The ternary radiometric image is a color composite map produced by modulating the red, green, and blue phosphors of the display device in proportion to the radiometric element concentration values of the potassium, thorium, and uranium data. Blue color was utilized to display the uranium channel, because it’s the noisiest channel and eye is less sensitive to variations in blue intensity. The blue colored zones are mainly very rich uranium, while the red and green zones are revealing higher contents of potassium and thorium, respectively. The white color on the ternary map indicates high concentrations of the potassium, thorium, and uranium elements. The dark colors zone indicates low concentrations of all the three elements. Lateral discontinuities of lithological units are revealed on the ternary radiometric map (Fig. 18) of the study area. This because the ternary map (Fig. 18) is very suitable for discriminating contacts between different lithologies. Hence, it is important to correlate the total gradient amplitude (TGA) lineaments with the ternary radiometric map (see Fig. 18). The TGA lineaments including Ifewara shear zone compliment the discontinuities on the ternary map. This reveals convergence of evidence from the aeromagnetic and radiometric data. In Addition, the 3-D Euler depth solutions were plotted on the ternary map (see Fig. 19) to correlate radiometric anomalies with the 3-D Euler depth solutions. A key observation on the ternary map (Fig. 19) is the white to light blue region adjacent the Ifewara shear zone which coincides with a region of very few 3-D Euler depth solutions. This indicates that the region has increased radiometric and decreased magnetic signals, which a very important sign of some regional mineralogical alterations. The consistence of results from aeromagnetic and radiometric methods gave important confidence that the TGA lineaments and 3-D Euler depth solutions have physical meaning in terms of the subsurface structural features.

### Spatial correlation of surface and subsurface lineaments

**Figure 20:**
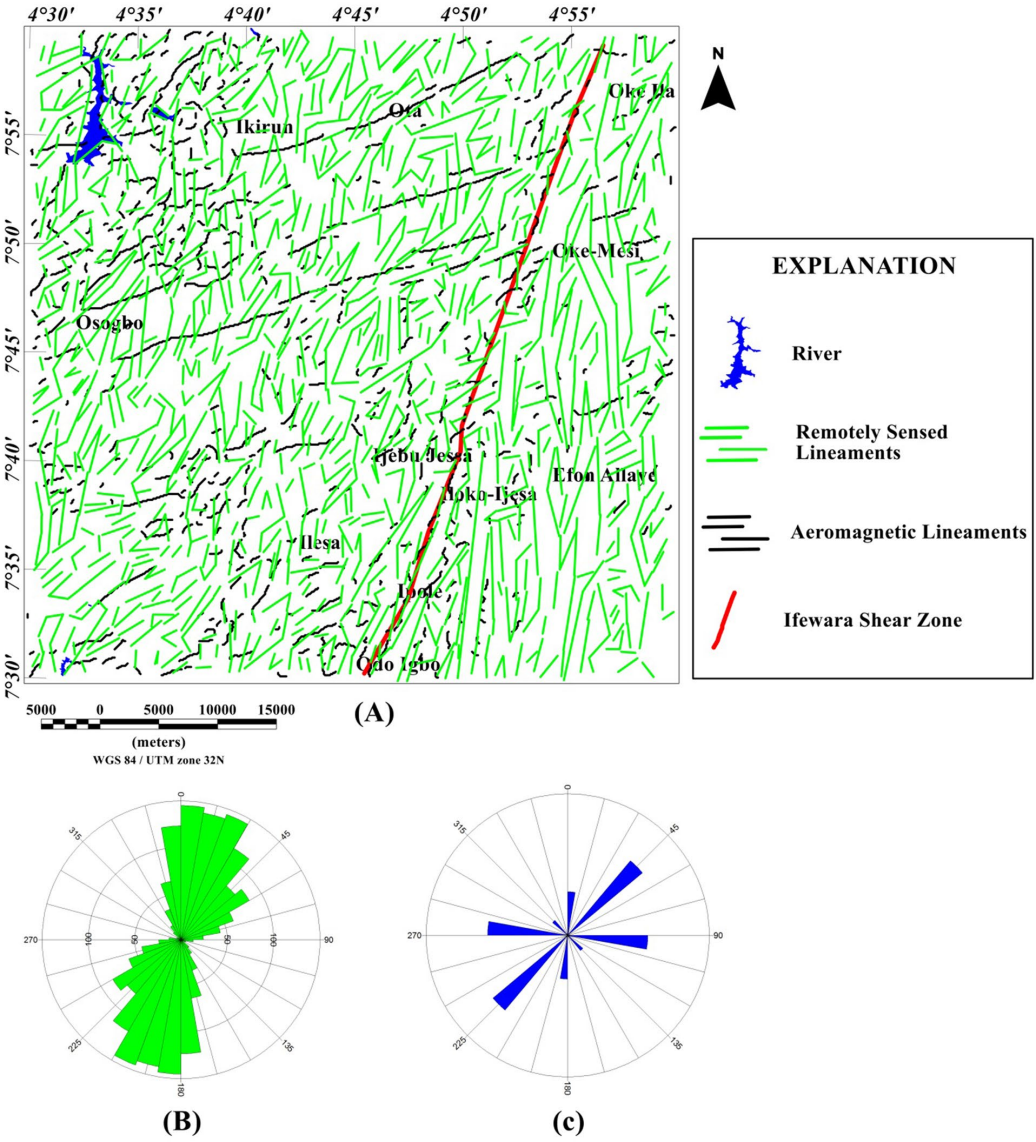
(**A**) Composite structural map of the Ife-Ilesha schist belt generated from the combined lineaments of SRTM (produced at 45° light angle at azimuths of 112.5°) and total gradient amplitude data. (**B**) Rose diagram of remotely sensed lineaments which offer trends patterns of surface lineaments. (**C**) Rose diagram of aeromagnetic lineaments which offer trends of subsurface lineaments. Note the extension of the Ifewara fault zone delineated from the total gradient amplitude data (indicated by as thick red line) coincides with some surface lineaments. Shuttle Radar Topography Mission image courtesy of the U.S. Geological Survey.

**Figure 21:**
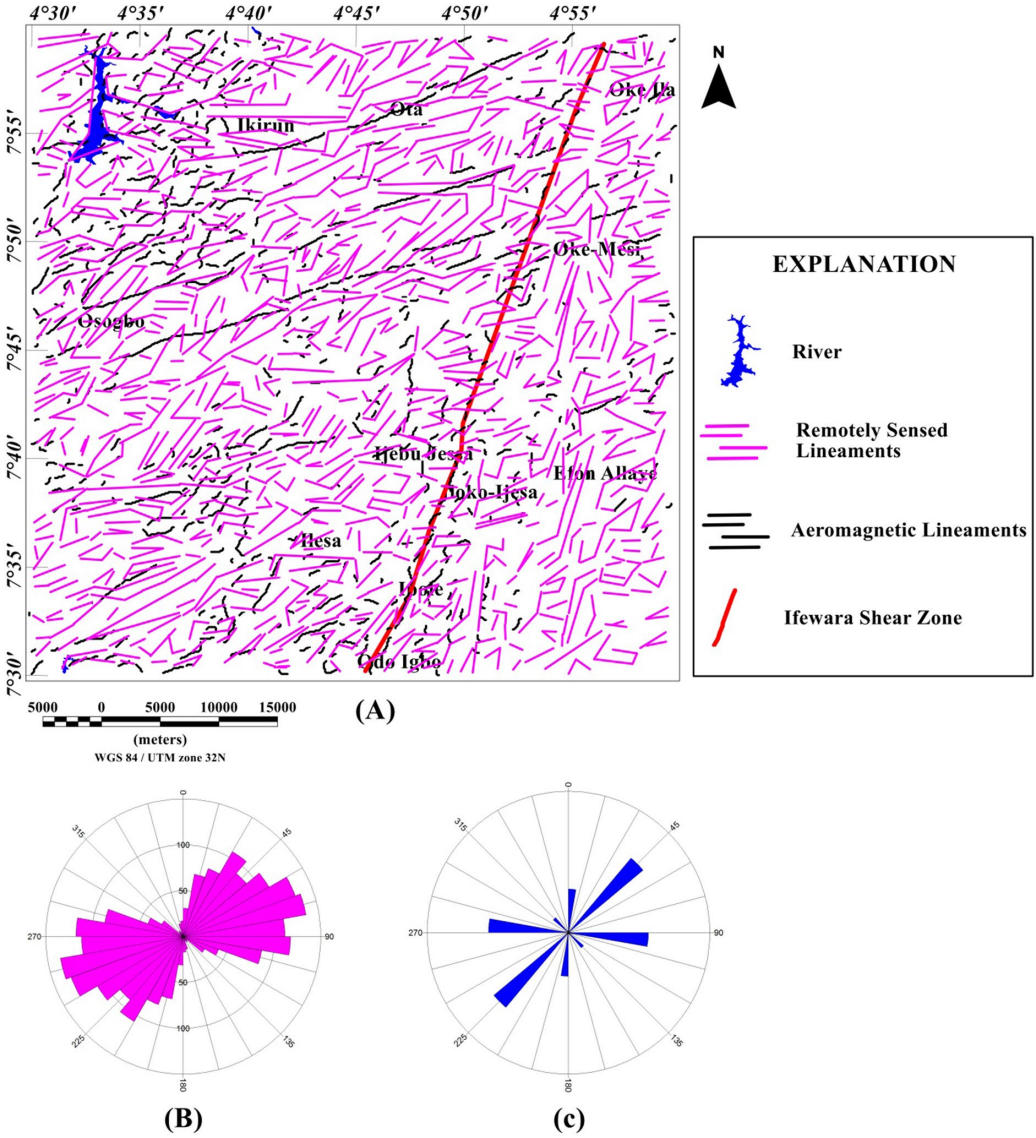
(**A**) Composite structural map of the Ife-Ilesha schist belt generated from the combined lineaments of SRTM (produced at 45° light angle at azimuths of 337.5°) and total gradient amplitude data. (**B**) Rose diagram of remotely sensed lineaments which offer trends patterns of surface lineaments. (**C**) Rose diagram of aeromagnetic lineaments which offer trends of subsurface lineaments. Note the extension of the Ifewara fault zone delineated from the total gradient amplitude data (indicated by as thick red line) coincides with some surface lineaments. Shuttle Radar Topography Mission image courtesy of the U.S. Geological Survey.

Lineaments were extracted from the shade-relief maps (Figs. 4, 5) and the extracted lineaments are shown in Figs. 20 and 21. Visual inspection of the extracted lineaments revealed the presence of a various morphological features attributed to faults, fractures and dykes. The Rose diagrams (Figs. 20B, 21B) of the extracted lineaments from the shaded-relief maps show that the study area is structured by N–S, NNE–SSW, NE–SW, ENE–WSW and E–W trending lineaments. Two composites lineament maps (Figs. 20, 21) of the Ife-Ilesha schist belt was produced from the superposition of surface lineaments extracted from Figs. 4 and 5 on the total gradient amplitude (TGA) lineaments in Fig. 9A. Several TGA lineaments on Figs. 20 and 21 follow the trends of the morphological features. This reveals that subsurface structural features produced the topographical features within the study area.

The visual integration of surface and subsurface Rose diagram (Figs. 20B,C, 21B,C) reveals similarity in trend directions of surface and subsurface lineaments. This close relationship is noticeable across the study area, including along the NNE-trending Ifewara shear zone. Based on the similarity of surface and subsurface lineament trends, the linear to curvilinear surface lineaments of the region probably represents the upward continuation of subsurface structural features. Hence, the evolution of the present day landform seems to be influenced by buried subsurface features such as intrusions, dykes and shear zones.

## Discussion

In this study, analysis of satellite, aeromagnetic and radiometric data was carried out to investigate the structurally controlled mineral deposit of the Ife-Ilesha schist belt. Utilizing the total gradient amplitude, ternary and shaded-relief maps, this study detected and visualized surface and subsurface structural features over a regional schist belt. Structural features such as intrusions, faults, fractures and shear zones are clearly detected within the study area. Structural trends vary across the Ife-Ilesha schist belt but are mainly in the NNE–SSW, ENE–WSW, N–S, NE–SW and E–W directions. Structural features within the region reveal several distinct magnetic anomalies which are due to depletion of magnetic minerals between adjacent magnetic rocks within the Ife-Ilesha schist belt. For example, the magnetic structural map (Fig. 9A) reveals an elongated NNE-trending lineament which shows similar geometric correlation with the Ifewara shear zone shown on the geological map of the study area (see Fig. 1). This lineament is also apparent on the 3-D Euler deconvolution map (Fig. 10). We interpret this elongated lineament as the important Ifewara shear zone crossing through the entire study area, with a minimum depth value of 90 m to the top of sources within the shear zone as revealed by the 3-D Euler deconvolution depth map. The aeromagnetic signature of Ifewara shear zone is closely linked with strain localization along contacts between competent rock units and most probably the alteration of basement rock units due to hydrothermal fluid flow in the later part of the Pan-African orogeny. The shear zone is also evidenced on the cross-section and total gradient amplitude maps (see Figs. 2, 8), with Fig. 2 revealing major aluminous quartzite units outcropping in ridges associated with the Ifewara shear zone. The superimposed aeromagnetic lineaments and 3-D Euler deconvolution depth solutions indicate the coincidence of Ifewara shear zone and distinct zones on the ternary map (see Fig. 19). This coincidence reveals high level of correlation of the shear zone between satellite, aeromagnetic and radiometric data. In addition, the Iperindo gold mining site mineralization which is located in Ife-Ilesha schist belt consists of a series of auriferous quartz-carbonate veins localized by subsidiary faults that are parallel to the main Ifewara shear^2^. Hence, from the superposition of the remotely sensed lineaments on the aeromagnetic lineament map has facilitated the confirmation of locations and continuations of near surface structural features. The delineated structures such as the Ifewara shear zone and subsidiary faults require further investigation for mineral deposits. Future studies involving the application of detailed mapping and high-resolution ground geophysical techniques are recommended to delineate infilling structural features related to rich mineral deposits.

New images are provided in this study to elucidate the evolution of structural features within the Ife-Ilesha schist belt. The remotely sensed imagery revealed remarkably morphological features and significant morphological differences can be observed. We reveal information about evolution of magnetic lineament with morphological features and we compare surface and subsurface features. The maxima of total gradient amplitude (TGA) are interpreted as subsurface structural features while 3-D Euler decovolution provides depth estimates of these structures. The depth information of the TGA lineaments can help miners target primary mineral deposits within the Ife-Ilesha schist belt. The results of the TGA technique coincide perfectly with remotely sensed imagery. Figure 9B reveals three main sets of lineament orientation which are the N–S, NE–SW and E–W trends, which have been entirely deduced using the high resolution aeromagnetic data. The N–S and NE–SW structural trend resulted from the compressional stress regime which dominates the Neoproterozoic. In addition, NW–SE structural trends are only slightly mapped in the studied region. The N–S, NNE–SSW, NE–SW, ENE–WSW and E–W structural trends are more prominent in the surface of the region as revealed by the remotely sensed rose diagrams (see Figs. 20B, 21B). The visual examination of the produced composite structural maps (Figs. 20A, 21A) allowed the interpretation that the subsurface structural features produce the topographical scraps within the region. The role of Pan-African orogeny in generating N–S and NE–SW structural features should be emphasized, in addition to the NNE–trending Ifewara fault zone. The intrusive magnetic sources within the Ifewara shear zone are also revealed by the 3-D Euler deconvolution map (Fig. 11). The NE–SW trending structural features northerly of the study area (Fig. 21) correlates with the regional scale structures on Fig. 3 (indicated with dashed thick blue lines) which host Sn–Nb–Ta-productive pegmatites^1,21^. By utilizing satellite and aeromagnetic data it is understood that most previous research turn a blind eye to many E–W trending structural features within the Ife-Ilesha schist belt. The E–W structures appear to have been produced in earlier orogeny events, consequent to the dominant Pan-African orogeny. Hence, the E–W trends will have important role to the pre-Pan-African orogeny.

## Conclusion

Satellite, aeromagnetic and radiometric data were utilized to map structural features associated with mineral deposits within the Ife-Ilesha schist belt. The results from the combined interpretation of these data indicate good correlation and also delineate new structural features within the region. Surface and subsurface expression of linear and curvilinear structures have been shown as prominent remotely sensed and aeromagnetic features. Composite structural maps produced from the total gradient amplitude (TGA) and shaded-relief maps reveal the Ifewara shear zone and other structures within the region. The depth estimates to these structural features are revealed using the 3-D Euler deconvolution technique. It is notable that the radiometric data evidently complement and facilitate the satellite and aeromagnetic data interpretations. For example, the Ifewara shear zone which was revealed by the TGA and hill-shading techniques coincidence with distinct pattern on the ternary map, indicating convergence of evidence from the satellite, aeromagnetic and radiometric data. Generally, there is a notable match between morphological features and subsurface lineaments within the Ife-Ilesha schist belt. This study presents mapped lineaments and their respective depth information, which can guide both explorationists and miners in the development of mineral deposits within the Ife-Ilesha schist belt.

## Data Availability

The Landsat-8 OLI/TIRS and Shuttle Radar Topography Mission data used for this study is publicly accessible and can be downloaded from the US Geological Survey (USGS) website. However, the high resolution aeromagnetic data is not publicly available, but can be acquired from the Nigeria Geological Survey Agency.
